# Coronavirus Disease 2019 Case Surveillance — United States, January 22–May 30, 2020

**DOI:** 10.15585/mmwr.mm6924e2

**Published:** 2020-06-19

**Authors:** Erin K. Stokes, Laura D. Zambrano, Kayla N. Anderson, Ellyn P. Marder, Kala M. Raz, Suad El Burai Felix, Yunfeng Tie, Kathleen E. Fullerton

**Affiliations:** 1 CDC COVID-19 Emergency Response.

The coronavirus disease 2019 (COVID-19) pandemic resulted in 5,817,385 reported cases and 362,705 deaths worldwide through May, 30, 2020,^†^ including 1,761,503 aggregated reported cases and 103,700 deaths in the United States.^§^ Previous analyses during February–early April 2020 indicated that age ≥65 years and underlying health conditions were associated with a higher risk for severe outcomes, which were less common among children aged <18 years (*1*–*3*). This report describes demographic characteristics, underlying health conditions, symptoms, and outcomes among 1,320,488 laboratory-confirmed COVID-19 cases individually reported to CDC during January 22–May 30, 2020. Cumulative incidence, 403.6 cases per 100,000 persons,^¶^ was similar among males (401.1) and females (406.0) and highest among persons aged ≥80 years (902.0). Among 599,636 (45%) cases with known information, 33% of persons were Hispanic or Latino of any race (Hispanic), 22% were non-Hispanic black (black), and 1.3% were non-Hispanic American Indian or Alaska Native (AI/AN). Among 287,320 (22%) cases with sufficient data on underlying health conditions, the most common were cardiovascular disease (32%), diabetes (30%), and chronic lung disease (18%). Overall, 184,673 (14%) patients were hospitalized, 29,837 (2%) were admitted to an intensive care unit (ICU), and 71,116 (5%) died. Hospitalizations were six times higher among patients with a reported underlying condition (45.4%) than those without reported underlying conditions (7.6%). Deaths were 12 times higher among patients with reported underlying conditions (19.5%) compared with those without reported underlying conditions (1.6%). The COVID-19 pandemic continues to be severe, particularly in certain population groups. These preliminary findings underscore the need to build on current efforts to collect and analyze case data, especially among those with underlying health conditions. These data are used to monitor trends in COVID-19 illness, identify and respond to localized incidence increase, and inform policies and practices designed to reduce transmission in the United States.

State and territorial health departments report daily aggregate counts of COVID-19 cases and deaths to CDC; these were tabulated according to date of report to examine reporting trends during January 22–May 30. In addition to aggregate counts, individual COVID-19 case reports were submitted via a CDC COVID-19 case report form** and the National Notifiable Diseases Surveillance System (NNDSS).^††^ Jurisdictions voluntarily report confirmed and probable^§§^ cases from reports submitted by health care providers and laboratories. A laboratory-confirmed COVID-19 case was defined as a person with a positive test result for SARS-CoV-2, the virus that causes COVID-19, from a respiratory specimen, using real-time reverse transcription–polymerase chain reaction testing. COVID-19 case data reported from 50 states, New York City, and the District of Columbia^¶¶^ were analyzed to examine reported demographic characteristics, underlying health conditions, clinical signs and symptoms, and severe outcomes, including hospitalization, ICU admission, and death. Data were missing for age, sex, and race or ethnicity in <1%, 1%, and 55% of reports, respectively.*** Cases reported without sex or age data were excluded from this analysis as were cases meeting only the probable case definition, along with persons repatriated to the United States from Wuhan, China, or the Diamond Princess cruise ship. Cumulative incidence was estimated using 2018 population estimates. Because of the high prevalence of missing race and ethnicity data, estimates of incidence and proportions of underlying health conditions, symptoms, and severe outcomes by race and ethnicity were not described. Analyses are descriptive and statistical comparisons were not performed.

CDC received notification of the first case of laboratory-confirmed COVID-19 in the United States on January 22, 2020.^†††^ As of May 30, an aggregate 1,761,503 U.S. COVID-19 cases and 103,700 deaths had been reported (Figure).^§§§^ The 7-day moving average number^¶¶¶^ of new daily cases peaked on April 12 (31,994) and deaths peaked on April 21 (2,856). As of May 30, the 7-day moving average numbers of new cases were 19,913 per day and deaths were 950 per day.

**FIGURE:**
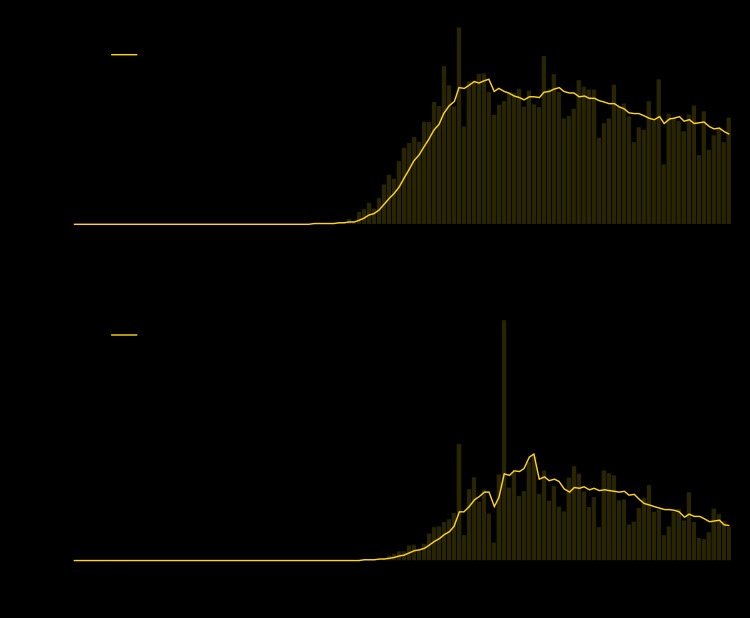
Daily number of COVID-19 cases^*^^,^^†^^,^^§^^,^^¶^ (A) and COVID-19–associated deaths** (B) reported to CDC — United States, January 22–May 30, 2020 **Abbreviation:** COVID-19 = coronavirus disease 2019. * From April 14, 2020, aggregate case counts reported by CDC included deaths attributable to both confirmed and probable COVID-19 as classified by reporting jurisdictions, using the Council of State and Territorial Epidemiologists position statement Interim-ID-20-01 (https://cdn.ymaws.com/www.cste.org/resource/resmgr/2020ps/interim-20-id-01_covid-19.pdf). ^†^ The upper quartile of the lag between onset date and reporting to CDC was 15 days. ^§^ The daily number of deaths reported by jurisdictions on April 14 includes 4,141 deaths newly classified as probable. ^¶^ Overall <1% of cases reported in aggregate to CDC were classified as probable. ** Overall 3.1% of deaths reported in aggregate to CDC were classified as occurring in persons with probable cases.

Among the 1,761,503 aggregate cases reported to CDC during January 22–May 30, individual case reports for 1,406,098 were submitted to CDC case surveillance. After exclusions, data for 1,320,488 (94%) cases were analyzed. Median age was 48 years (interquartile range = 33–63 years). Incidence was 403.6 cases per 100,000 population (Table 1) and was similar among females (406.0) and males (401.1).**** Incidence was higher among persons aged 40–49 years (541.6) and 50–59 years (550.5) than among those aged 60–69 years (478.4) and 70–79 years (464.2). Incidence was highest among persons aged ≥80 years (902.0)^††††^ and lowest among children aged ≤9 years (51.1). Among the 599,636 (45%) cases with information on both race and ethnicity, 36% of persons were non-Hispanic white, 33% were Hispanic, 22% were black, 4% were non-Hispanic Asian, 4% were non-Hispanic, other or multiple race, 1.3% were AI/AN, and <1% were non-Hispanic Native Hawaiian or other Pacific Islander.

**TABLE 1:** Reported laboratory-confirmed COVID-19 cases and estimated cumulative incidence,* by sex^†^ and age group — United States, January 22–May 30, 2020

Age group (yrs)	Males		Females		Total	
	No. (%)	Cumulative incidence*	No. (%)	Cumulative incidence*	No. (%)	Cumulative incidence*
0–9	10,743 (1.7)	52.5	9,715 (1.4)	49.7	20,458 (1.5)	51.1
10–19	24,302 (3.8)	113.4	24,943 (3.7)	121.4	49,245 (3.7)	117.3
20–29	85,913 (13.3)	370.0	96,556 (14.3)	434.6	182,469 (13.8)	401.6
30–39	108,319 (16.8)	492.8	106,530 (15.8)	490.5	214,849 (16.3)	491.6
40–49	109,745 (17.0)	547.0	109,394 (16.2)	536.2	219,139 (16.6)	541.6
50–59	119,152 (18.4)	568.8	116,622 (17.3)	533.0	235,774 (17.9)	550.5
60–69	93,596 (14.5)	526.9	85,411 (12.7)	434.6	179,007 (13.6)	478.4
70–79	53,194 (8.2)	513.7	52,058 (7.7)	422.7	105,252 (8.0)	464.2
≥80	41,394 (6.4)	842.0	72,901 (10.8)	940.0	114,295 (8.7)	902.0
**All ages**	**646,358 (100.0)**	**401.1**	**674,130 (100.0)**	**406.0**	**1,320,488 (100.0)**	**403.6**

**Abbreviation:** COVID-19 = coronavirus disease 2019.

* Per 100,000 population.

**^†^** The analytic dataset excludes cases reported through case surveillance that were missing information on sex (n = 19,918) or age (n = 2,379).

Symptom status (symptomatic versus asymptomatic) was reported for 616,541 (47%) cases; among these, 22,007 (4%) were asymptomatic. Among 373,883 (28%) cases with data on individual symptoms, 70% noted fever, cough, or shortness of breath; 36% reported muscle aches, and 34% reported headache (Table 2). Overall, 31,191 (8%) persons reported loss of smell or taste.^§§§§^ Among patients aged ≥80 years, 60% reported fever, cough, or shortness of breath. No other symptoms were reported by >10% of persons in this age group.

**TABLE 2:** Reported underlying health conditions* and symptoms^†^ among persons with laboratory-confirmed COVID-19, by sex and age group — United States, January 22–May 30, 2020

Characteristic	No. (%)											
	Total	Sex		Age group (yrs)								
		Male	Female	≤9	10–19	20–29	30–39	40–49	50–59	60–69	70–79	≥80
**Total population**	**1,320,488**	**646,358**	**674,130**	**20,458**	**49,245**	**182,469**	**214,849**	**219,139**	**235,774**	**179,007**	**105,252**	**114,295**
**Underlying health condition^§^**												
Known underlying medical condition status*	**287,320 (21.8)**	138,887 (21.5)	148,433 (22.0)	2,896 (14.2)	7,123 (14.5)	27,436 (15.0)	33,483 (15.6)	40,572 (18.5)	54,717 (23.2)	50,125 (28.0)	34,400 (32.7)	36,568 (32.0)
Any cardiovascular disease^¶^	**92,546 (32.2)**	47,567 (34.2)	44,979 (30.3)	78 (2.7)	164 (2.3)	1,177 (4.3)	3,588 (10.7)	8,198 (20.2)	16,954 (31.0)	21,466 (42.8)	18,763 (54.5)	22,158 (60.6)
Any chronic lung disease	**50,148 (17.5)**	20,930 (15.1)	29,218 (19.7)	363 (12.5)	1,285 (18)	4,537 (16.5)	5,110 (15.3)	6,127 (15.1)	8,722 (15.9)	9,200 (18.4)	7,436 (21.6)	7,368 (20.1)
Renal disease	**21,908 (7.6)**	12,144 (8.7)	9,764 (6.6)	21 (0.7)	34 (0.5)	204 (0.7)	587 (1.8)	1,273 (3.1)	2,789 (5.1)	4,764 (9.5)	5,401 (15.7)	6,835 (18.7)
Diabetes	**86,737 (30.2)**	45,089 (32.5)	41,648 (28.1)	12 (0.4)	225 (3.2)	1,409 (5.1)	4,106 (12.3)	9,636 (23.8)	19,589 (35.8)	22,314 (44.5)	16,594 (48.2)	12,852 (35.1)
Liver disease	**3,953 (1.4)**	2,439 (1.8)	1,514 (1.0)	5 (0.2)	19 (0.3)	132 (0.5)	390 (1.2)	573 (1.4)	878 (1.6)	1,074 (2.1)	583 (1.7)	299 (0.8)
Immunocompromised	**15,265 (5.3)**	7,345 (5.3)	7,920 (5.3)	61 (2.1)	146 (2.0)	646 (2.4)	1,253 (3.7)	2,005 (4.9)	3,190 (5.8)	3,421 (6.8)	2,486 (7.2)	2,057 (5.6)
Neurologic/Neurodevelopmental disability	**13,665 (4.8)**	6,193 (4.5)	7,472 (5.0)	41 (1.4)	113 (1.6)	395 (1.4)	533 (1.6)	734 (1.8)	1,338 (2.4)	2,006 (4.0)	2,759 (8.0)	5,746 (15.7)
**Symptom^§^**												
Known symptom status^†^	**373,883 (28.3)**	178,223 (27.6)	195,660 (29.0)	5,188 (25.4)	12,689 (25.8)	51,464 (28.2)	59,951 (27.9)	62,643 (28.6)	70,040 (29.7)	52,178 (29.1)	28,583 (27.2)	31,147 (27.3)
Fever, cough, or shortness of breath	**260,706 (69.7)**	125,768 (70.6)	134,938 (69.0)	3,278 (63.2)	7,584 (59.8)	35,072 (68.1)	42,016 (70.1)	45,361 (72.4)	51,283 (73.2)	37,701 (72.3)	19,583 (68.5)	18,828 (60.4)
Fever**^††^**	**161,071 (43.1)**	80,578 (45.2)	80,493 (41.1)	2,404 (46.3)	4,443 (35.0)	20,381 (39.6)	25,887 (43.2)	28,407 (45.3)	32,375 (46.2)	23,591 (45.2)	12,190 (42.6)	11,393 (36.6)
Cough	**187,953 (50.3)**	89,178 (50.0)	98,775 (50.5)	1,912 (36.9)	5,257 (41.4)	26,284 (51.1)	31,313 (52.2)	34,031 (54.3)	38,305 (54.7)	27,150 (52.0)	12,837 (44.9)	10,864 (34.9)
Shortness of breath	**106,387 (28.5)**	49,834 (28.0)	56,553 (28.9)	339 (6.5)	2,070 (16.3)	13,649 (26.5)	16,851 (28.1)	18,978 (30.3)	21,327 (30.4)	16,018 (30.7)	8,971 (31.4)	8,184 (26.3)
Myalgia	**135,026 (36.1)**	61,922 (34.7)	73,104 (37.4)	537 (10.4)	3,737 (29.5)	21,153 (41.1)	26,464 (44.1)	28,064 (44.8)	28,594 (40.8)	17,360 (33.3)	6,015 (21.0)	3,102 (10.0)
Runny nose	**22,710 (6.1)**	9,900 (5.6)	12,810 (6.5)	354 (6.8)	1,025 (8.1)	4,591 (8.9)	4,406 (7.3)	4,141 (6.6)	4,100 (5.9)	2,671 (5.1)	923 (3.2)	499 (1.6)
Sore throat	**74,840 (20.0)**	31,244 (17.5)	43,596 (22.3)	664 (12.8)	3,628 (28.6)	14,493 (28.2)	14,855 (24.8)	14,490 (23.1)	13,930 (19.9)	8,192 (15.7)	2,867 (10.0)	1,721 (5.5)
Headache	**128,560 (34.4)**	54,721 (30.7)	73,839 (37.7)	785 (15.1)	5,315 (41.9)	23,723 (46.1)	26,142 (43.6)	26,245 (41.9)	26,057 (37.2)	14,735 (28.2)	4,163 (14.6)	1,395 (4.5)
Nausea/Vomiting	**42,813 (11.5)**	16,549 (9.3)	26,264 (13.4)	506 (9.8)	1,314 (10.4)	6,648 (12.9)	7,661 (12.8)	8,091 (12.9)	8,737 (12.5)	5,953 (11.4)	2,380 (8.3)	1,523 (4.9)
Abdominal pain	**28,443 (7.6)**	11,553 (6.5)	16,890 (8.6)	349 (6.7)	978 (7.7)	4,211 (8.2)	5,150 (8.6)	5,531 (8.8)	6,134 (8.8)	3,809 (7.3)	1,449 (5.1)	832 (2.7)
Diarrhea	**72,039 (19.3)**	32,093 (18.0)	39,946 (20.4)	704 (13.6)	1,712 (13.5)	9,867 (19.2)	12,769 (21.3)	13,958 (22.3)	15,536 (22.2)	10,349 (19.8)	4,402 (15.4)	2,742 (8.8)
Loss of smell or taste	**31,191 (8.3)**	12,717 (7.1)	18,474 (9.4)	67 (1.3)	1,257 (9.9)	6,828 (13.3)	6,907 (11.5)	6,361 (10.2)	5,828 (8.3)	2,930 (5.6)	775 (2.7)	238 (0.8)

**Abbreviation**: COVID-19 = coronavirus disease 2019.

* Status of underlying health conditions known for 287,320 persons. Status was classified as “known” if any of the following conditions were reported as present or absent: diabetes mellitus, cardiovascular disease (including hypertension), severe obesity (body mass index ≥40 kg/m^2^), chronic renal disease, chronic liver disease, chronic lung disease, immunocompromising condition, autoimmune condition, neurologic condition (including neurodevelopmental, intellectual, physical, visual, or hearing impairment), psychologic/psychiatric condition, and other underlying medical condition not otherwise specified.

^†^ Symptom status was known for 373,883 persons. Status was classified as “known” if any of the following symptoms were reported as present or absent: fever (measured >100.4°F [38°C] or subjective), cough, shortness of breath, wheezing, difficulty breathing, chills, rigors, myalgia, rhinorrhea, sore throat, chest pain, nausea or vomiting, abdominal pain, headache, fatigue, diarrhea (≥3 loose stools in a 24-hour period), or other symptom not otherwise specified on the form.

^§^ Responses include data from standardized fields supplemented with data from free-text fields. Information for persons with loss of smell or taste was exclusively extracted from a free-text field; therefore, persons exhibiting this symptom were likely underreported.

^¶^ Includes persons with reported hypertension.

** Includes all persons with at least one of these symptoms reported.

**^††^** Persons were considered to have a fever if information on either measured or subjective fever variables if “yes” was reported for either variable.

Among 287,320 (22%) cases with data on individual underlying health conditions, those most frequently reported were cardiovascular disease (32%), diabetes (30%), and chronic lung disease (18%) (Table 2); the reported proportions were similar among males and females. The frequency of conditions reported varied by age group: cardiovascular disease was uncommon among those aged ≤39 years but was reported in approximately half of the cases among persons aged ≥70 years. Among 63,896 females aged 15–44 years with known pregnancy status, 6,708 (11%) were reported to be pregnant.

Among the 1,320,488 cases, outcomes for hospitalization, ICU admission, and death were available for 46%, 14%, and 36%, respectively. Overall, 184,673 (14%) patients were hospitalized, including 29,837 (2%) admitted to the ICU; 71,116 (5%) patients died (Table 3). Severe outcomes were more commonly reported for patients with reported underlying conditions. Hospitalizations were six times higher among patients with a reported underlying condition than those without reported underlying conditions (45.4% versus 7.6%). Deaths were 12 times higher among patients with reported underlying conditions compared with those without reported underlying conditions (19.5% versus 1.6%). The percentages of males who were hospitalized (16%), admitted to the ICU (3%), and who died (6%) were higher than were those for females (12%, 2%, and 5%, respectively). The percentage of ICU admissions was highest among persons with reported underlying conditions aged 60–69 years (11%) and 70–79 years (12%). Death was most commonly reported among persons aged ≥80 years regardless of the presence of underlying conditions (with underlying conditions 50%; without 30%).

**TABLE 3:** Reported hospitalizations,*^,^**^†^** intensive care unit (ICU) admissions,**^§^** and deaths**^¶^** among laboratory-confirmed COVID-19 patients with and without reported underlying health conditions,** by sex and age — United States, January 22–May 30, 2020

Characteristic (no.)	Outcome, no./total no. (%)^††^								
	Reported hospitalizations*^,†^ (including ICU)			Reported ICU admission^§^			Reported deaths^¶^		
	Among all patients	Among patients with reported underlying health conditions	Among patients with no reported underlying health conditions	Among all patients	Among patients with reported underlying health conditions	Among patients with no reported underlying health conditions	Among all patients	Among patients with reported underlying health conditions	Among patients with no reported underlying health conditions
**Sex**									
Male (646,358)	101,133/646,358 (15.6)	49,503/96,839 (51.1)	3,596/42,048 (8.6)	18,394/646,358 (2.8)	10,302/96,839 (10.6)	864/42,048 (2.1)	38,773/646,358 (6.0)	21,667/96,839 (22.4)	724/42,048 (1.7)
Female (674,130)	83,540/674,130 (12.4)	40,698/102,040 (39.9)	3,087/46,393 (6.7)	11,443/674,130 (1.7)	6,672/102,040 (6.5)	479/46,393 (1.0)	32,343/674,130 (4.8)	17,145/102,040 (16.8)	707/46,393 (1.5)
**Age group (yrs)**									
≤9 (20,458)	848/20,458 (4.1)	138/619 (22.3)	84/2,277 (3.7)	141/20,458 (0.7)	31/619 (5.0)	16/2,277 (0.7)	13/20,458 (0.1)	4/619 (0.6)	2/2,277 (0.1)
10–19 (49,245)	1,234/49,245 (2.5)	309/2,076 (14.9)	115/5,047 (2.3)	216/49,245 (0.4)	72/2,076 (3.5)	17/5,047 (0.3)	33/49,245 (0.1)	16/2,076 (0.8)	4/5,047 (0.1)
20–29 (182,469)	6,704/182,469 (3.7)	1,559/8,906 (17.5)	498/18,530 (2.7)	864/182,469 (0.5)	300/8,906 (3.4)	56/18,530 (0.3)	273/182,469 (0.1)	122/8,906 (1.4)	24/18,530 (0.1)
30–39 (214,849)	12,570/214,849 (5.9)	3,596/14,854 (24.2)	828/18,629 (4.4)	1,879/214,849 (0.9)	787/14,854 (5.3)	135/18,629 (0.7)	852/214,849 (0.4)	411/14,854 (2.8)	21/18,629 (0.1)
40–49 (219,139)	19,318/219,139 (8.8)	7,151/24,161 (29.6)	1,057/16,411 (6.4)	3,316/219,139 (1.5)	1,540/24,161 (6.4)	208/16,411 (1.3)	2,083/219,139 (1.0)	1,077/24,161 (4.5)	58/16,411 (0.4)
50–59 (235,774)	31,588/235,774 (13.4)	14,639/40,297 (36.3)	1,380/14,420 (9.6)	5,986/235,774 (2.5)	3,335/40,297 (8.3)	296/14,420 (2.1)	5,639/235,774 (2.4)	3,158/40,297 (7.8)	131/14,420 (0.9)
60–69 (179,007)	39,422/179,007 (22.0)	21,064/42,206 (49.9)	1,216/7,919 (15.4)	7,403/179,007 (4.1)	4,588/42,206 (10.9)	291/7,919 (3.7)	11,947/179,007 (6.7)	7,050/42,206 (16.7)	187/7,919 (2.4)
70–79 (105,252)	35,844/105,252 (34.1)	20,451/31,601 (64.7)	780/2,799 (27.9)	5,939/105,252 (5.6)	3,771/31,601 (11.9)	199/2,799 (7.1)	17,510/105,252 (16.6)	10,008/31,601 (31.7)	286/2,799 (10.2)
≥80 (114,295)	37,145/114,295 (32.5)	21,294/34,159 (62.3)	725/2,409 (30.1)	4,093/114,295 (3.6)	2,550/34,159 (7.5)	125/2,409 (5.2)	32,766/114,295 (28.7)	16,966/34,159 (49.7)	718/2,409 (29.8)
**Total (1,320,488)**	**184,673/1,320,488 (14.0)**	**90,201/198,879 (45.4)**	**6,683/88,441 (7.6)**	**29,837/1,320,488 (2.3)**	**16,974/198,879 (8.5)**	**1,343/88,441 (1.5)**	**71,116/1,320,488 (5.4)**	**38,812/198,879 (19.5)**	**1,431/88,441 (1.6)**

**Abbreviation:** COVID-19 = coronavirus disease 2019.

* Hospitalization status was known for 600,860 (46%). Among 184,673 hospitalized patients, the presence of underlying health conditions was known for 96,884 (53%).

^†^ Includes reported ICU admissions.

^§^ ICU admission status was known for 186,563 (14%) patients among the total case population, representing 34% of hospitalized patients. Among 29,837 patients admitted to the ICU, the status of underlying health conditions was known for 18,317 (61%).

^¶^ Death outcomes were known for 480,565 (36%) patients. Among 71,116 reported deaths through case surveillance, the status of underlying health conditions was known for 40,243 (57%) patients.

** Status of underlying health conditions was known for 287,320 (22%) patients. Status was classified as “known” if any of the following conditions were noted as present or absent: diabetes mellitus, cardiovascular disease including hypertension, severe obesity body mass index ≥40 kg/m^2^, chronic renal disease, chronic liver disease, chronic lung disease, any immunocompromising condition, any autoimmune condition, any neurologic condition including neurodevelopmental, intellectual, physical, visual, or hearing impairment, any psychologic/psychiatric condition, and any other underlying medical condition not otherwise specified.

^††^ Outcomes were calculated as the proportion of persons reported to be hospitalized, admitted to an ICU, or who died among total in the demographic group. Outcome underreporting could result from outcomes that occurred but were not reported through national case surveillance or through clinical progression to severe outcomes that occurred after time of report.

## Discussion

As of May 30, a total of 1,761,503 aggregate U.S. cases of COVID-19 and 103,700 associated deaths were reported to CDC. Although average daily reported cases and deaths are declining, 7-day moving averages of daily incidence of COVID-19 cases indicate ongoing community transmission.^¶¶¶¶^

The COVID-19 case data summarized here are essential statistics for the pandemic response and rely on information systems developed at the local, state, and federal level over decades for communicable disease surveillance that were rapidly adapted to meet an enormous, new public health threat. CDC aggregate counts are consistent with those presented through the Johns Hopkins University (JHU) Coronavirus Resource Center, which reported a cumulative total of 1,770,165 U.S. cases and 103,776 U.S. deaths on May 30, 2020.***** Differences in aggregate counts between CDC and JHU might be attributable to differences in reporting practices to CDC and jurisdictional websites accessed by JHU.

Reported cumulative incidence in the case surveillance population among persons aged ≥20 years is notably higher than that among younger persons. The lower incidence in persons aged ≤19 years could be attributable to undiagnosed milder or asymptomatic illnesses among this age group that were not reported. Incidence in persons aged ≥80 years was nearly double that in persons aged 70–79 years.

Among cases with known race and ethnicity, 33% of persons were Hispanic, 22% were black, and 1.3% were AI/AN. These findings suggest that persons in these groups, who account for 18%, 13%, and 0.7% of the U.S. population, respectively, are disproportionately affected by the COVID-19 pandemic. The proportion of missing race and ethnicity data limits the conclusions that can be drawn from descriptive analyses; however, these findings are consistent with an analysis of COVID-19–Associated Hospitalization Surveillance Network (COVID-NET)^†††††^ data that found higher proportions of black and Hispanic persons among hospitalized COVID-19 patients than were in the overall population (*4*). The completeness of race and ethnicity variables in case surveillance has increased from 20% to >40% from April 2 to June 2. Although reporting of race and ethnicity continues to improve, more complete data might be available in aggregate on jurisdictional websites or through sources like the COVID Tracking Project’s COVID Racial Data Tracker.^§§§§§^

The data in this report show that the prevalence of reported symptoms varied by age group but was similar among males and females. Fewer than 5% of persons were reported to be asymptomatic when symptom data were submitted. Persons without symptoms might be less likely to be tested for COVID-19 because initial guidance recommended testing of only symptomatic persons and was hospital-based. Guidance on testing has evolved throughout the response.^¶¶¶¶¶^ Whereas incidence among males and females was similar overall, severe outcomes were more commonly reported among males. Prevalence of reported severe outcomes increased with age; the percentages of hospitalizations, ICU admissions, and deaths were highest among persons aged ≥70 years, regardless of underlying conditions, and lowest among those aged ≤19 years. Hospitalizations were six times higher and deaths 12 times higher among those with reported underlying conditions compared with those with none reported. These findings are consistent with previous reports that found that severe outcomes increased with age and underlying condition, and males were hospitalized at a higher rate than were females (*2**,**4*,*5*).

The findings in this report are subject to at least three limitations. First, case surveillance data represent a subset of the total cases of COVID-19 in the United States; not every case in the community is captured through testing and information collected might be limited if persons are unavailable or unwilling to participate in case investigations or if medical records are unavailable for data extraction. Reported cumulative incidence, although comparable across age and sex groups within the case surveillance population, are underestimates of the U.S. cumulative incidence of COVID-19. Second, reported frequencies of individual symptoms and underlying health conditions presented from case surveillance likely underestimate the true prevalence because of missing data. Finally, asymptomatic cases are not captured well in case surveillance. Asymptomatic persons are unlikely to seek testing unless they are identified through active screening (e.g., contact tracing), and, because of limitations in testing capacity and in accordance with guidance, investigation of symptomatic persons is prioritized. Increased identification and reporting of asymptomatic cases could affect patterns described in this report.

Similar to earlier reports on COVID-19 case surveillance, severe outcomes were more commonly reported among persons who were older and those with underlying health conditions (*1*). Findings in this report align with demographic and severe outcome trends identified through COVID-NET (*4*). Findings from case surveillance are evaluated along with enhanced surveillance data and serologic survey results to provide a comprehensive picture of COVID-19 trends, and differences in proportion of cases by racial and ethnic groups should continue to be examined in enhanced surveillance to better understand populations at highest risk.

Since the U.S. COVID-19 response began in January, CDC has built on existing surveillance capacity to monitor the impact of illness nationally. Collection of detailed case data is a resource-intensive public health activity, regardless of disease incidence. The high incidence of COVID-19 has highlighted limitations of traditional public health case surveillance approaches to provide real-time intelligence and supports the need for continued innovation and modernization. Despite limitations, national case surveillance of COVID-19 serves a critical role in the U.S. COVID-19 response: these data demonstrate that the COVID-19 pandemic is an ongoing public health crisis in the United States that continues to affect all populations and result in severe outcomes including death. National case surveillance findings provide important information for targeted enhanced surveillance efforts and development of interventions critical to the U.S. COVID-19 response.

**Summary**

**What is already known about this topic?**

Surveillance data reported to CDC through April 2020 indicated that COVID-19 leads to severe outcomes in older adults and those with underlying health conditions.

**What is added by this report?**

As of May 30, 2020, among COVID-19 cases, the most common underlying health conditions were cardiovascular disease (32%), diabetes (30%), and chronic lung disease (18%). Hospitalizations were six times higher and deaths 12 times higher among those with reported underlying conditions compared with those with none reported.

**What are the implications for public health practice?**

Surveillance at all levels of government, and its continued modernization, is critical for monitoring COVID-19 trends and identifying groups at risk for infection and severe outcomes. These findings highlight the continued need for community mitigation strategies, especially for vulnerable populations, to slow COVID-19 transmission.

## Footnotes

* These authors contributed equally to this report.

† https://www.who.int/emergencies/diseases/novel-coronavirus-2019/situation-reports.

§ CDC official counts of cases and deaths, released daily on https://www.cdc.gov/coronavirus/2019-ncov/cases-updates/cases-in-us.html, are aggregate counts from reporting jurisdictions. Throughout the COVID-19 pandemic, CDC has been tracking both aggregate and individual (i.e., line-list) counts of cases and deaths. For aggregate counts, from January 22 to March 2, 2020, CDC provided laboratory confirmation for all U.S. confirmed cases. Starting March 3, jurisdiction partners validated aggregate counts each night for report out at 12 p.m. the following day by CDC. For individual counts, jurisdiction partners electronically submit standardized information for individual cases of COVID-19 to CDC. From April 14, aggregate and individual counts included confirmed and probable cases and deaths, according to the Council of State and Territorial Epidemiologists position statement Interim 20-ID-01 (https://cdn.ymaws.com/www.cste.org/resource/resmgr/2020ps/interim-20-id-01_covid-19.pdf; https://wwwn.cdc.gov/nndss/conditions/coronavirus-disease-2019-covid-19/case-definition/2020/).

¶ Incidence was calculated per 100,000 population using 2018 U.S. Census population estimates for U.S. states and the District of Columbia obtained from CDC WONDER (https://wonder.cdc.gov/single-race-population.html).

** https://www.cdc.gov/coronavirus/2019-ncov/php/reporting-pui.html.

†† https://wwwn.cdc.gov/nndss; https://wwwn.cdc.gov/nndss/covid-19-response.html.

§§ According to the Council of State and Territorial Epidemiologists position statement Interim 20-ID-01, a probable case must 1) meet clinical criteria and epidemiologic criteria with no confirmatory laboratory testing performed; 2) have presumptive laboratory evidence, including detection of specific antigen or antibody in a clinical specimen, and meet clinical criteria or epidemiologic criteria; or 3) meet vital records criteria with no confirmatory laboratory testing performed. (https://cdn.ymaws.com/www.cste.org/resource/resmgr/2020ps/interim-20-id-01_covid-19.pdf)

¶¶ Cases reported from U.S. territories were not included in the analysis because of limited case reporting and lack of available demographically stratified census data. Cases excluded from this analysis include those reported from Guam (116), the Northern Mariana Islands (16), Puerto Rico (one), and the U.S. Virgin Islands (71).

*** Cases reported as Hispanic were categorized as “Hispanic or Latino persons of any race” regardless of availability of race data.

††† The first laboratory-confirmed case of COVID-19 in the United States was confirmed on January 20, 2020, and reported to CDC on January 22, 2020. The upper quartile of the lag between onset date and reporting to CDC was 15 days.

§§§ From April 15 to May 30, 2020, these aggregate counts include both confirmed and probable cases and deaths. Overall, <1% of cases and 3.1% of deaths were classified as probable.

¶¶¶ The 7-day moving average of new cases and deaths (current day + 6 preceding days / 7) was calculated to smooth expected variations in daily counts.

**** In some age groups, males had higher incidence, and in some age groups, females had higher incidence.

†††† Among those aged ≥85 years, incidence was 1,138 per 100,000.

§§§§ Responses include data from standardized fields supplemented with data from free-text fields; therefore, persons exhibiting this symptom might be underreported.

¶¶¶¶ Community transmission is defined by states and reflects varying conditions at the local and state levels.

***** COVID-19 Dashboard by the Center for Systems Science and Engineering at Johns Hopkins University is a publicly available data tracker that extracts data from state, territorial, and local public health websites (https://coronavirus.jhu.edu/us-map). Data are archived in GitHub (https://github.com/CSSEGISandData/COVID-19/blob/master/csse_covid_19_data/csse_covid_19_daily_reports_us/05-30-2020.csv).

††††† COVID-Net is a population-based surveillance system that collects data on laboratory-confirmed COVID-19–associated hospitalizations (https://www.cdc.gov/coronavirus/2019-ncov/covid-data/covid-net/purpose-methods.html).

§§§§§ The COVID Tracking Project is *The Atlantic’s* volunteer organization to collect and publish U.S. COVID-19 data (https://covidtracking.com/race/dashboard).

¶¶¶¶¶ https://www.cdc.gov/coronavirus/2019-ncov/symptoms-testing/testing.html.
